# Molecular prevalence and genotypes of *Enterocytozoon bieneusi* in cancer patients under chemotherapy in Aegean region of Türkiye

**DOI:** 10.1186/s12866-024-03369-3

**Published:** 2024-06-26

**Authors:** Ayşegül Aksoy Gökmen, Tülay Öncü Öner, Sedef Erkunt Alak, Ecem Su Koçkaya, Mervenur Güvendi, Mehmet Karabey, Ahmet Alacacıoğlu, Bayram Pektaş, Aysu Değirmenci Döşkaya, Muhammet Karakavuk, Mert Döşkaya, Cemal Ün, Adnan Yüksel Gürüz, Selçuk Kaya, Hüseyin Can

**Affiliations:** 1 Faculty of Medicine, Department of Medical Microbiology, İzmir Katip Çelebi University, İzmir, Türkiye; 2 Faculty of Engineering, Department of Bioengineering, Manisa Celal Bayar University, Manisa, Türkiye; 3 Vaccine Development Application and Research Center, Ege University, İzmir, Türkiye; 4 Faculty of Science, Department of Biology, Molecular Biology Section, Ege University, İzmir, Türkiye; 5 Department of Medical Virology, Basaksehir Çam and Sakura City Hospital, University of Health Science, İstanbul, Türkiye; 6 Faculty of Medicine, Department of Internal Medicine, İzmir Katip Çelebi University, İzmir, Türkiye; 7 Department of Microbiology, İzmir Atatürk Training and Research Hospital, İzmir, Türkiye; 8 Faculty of Medicine, Department of Parasitology, Ege University, İzmir, Türkiye; 9 Department of Vaccine Studies, Institute of Health Sciences, Ege University, İzmir, Türkiye; 10 Ödemiş Vocational High School, Ege University, İzmir, Türkiye

**Keywords:** *E. bieneusi*, Cancer patients, Real time PCR, Genotyping

## Abstract

**Background:**

*Enterocytozoon bieneusi* is the most common species found in humans. Although *E. bieneusi* has been investigated in humans, genotype profile of *E. bieneusi* is not known in Türkiye.

**Methods:**

In this study, we screened *E. bieneusi* in patients (*n* = 94) with different types of malignant solid tumors by Real Time PCR and then sequenced *E. bieneusi* positive samples. All cancer patients were undergoing chemotherapy and had diarrhea. Moreover, as control groups, we also screened *E. bieneusi* in patients with diarrhea (*n* = 50) and without diarrhea (*n* = 50).

**Results:**

Among all patients analyzed, 33 (17%) were found to be *E. bieneusi*-positive. As the patients were categorized, the molecular prevalence of *E. bieneusi* increased to 25.5% among cancer patients with diarrhea. However, the molecular prevalence of *E. bieneusi* was found to be lower in patients with presenting only diarrhea (8%) and patients without diarrhea (10%). The high molecular prevalence value detected among cancer patients with diarrhea was also statistically significant compared to other patient groups (P = 0.00112 and P = 0.0269). Among the 33 Real Time PCR positive samples, 10 of them were amplified by nested PCR and among these 10 samples, 6 of them were successfully genotyped. The phylogenetic tree showed the presence of D and Type IV which were also identified in stray cats living in İzmir in our previous study.

**Conclusions:**

High molecular prevalence value indicates the importance of screening stool samples of cancer patients with diarrhea for *E. bieneusi* and genotyping results indicate that D and Type IV are circulating between humans and cats.

## Background

Microsporidia species are obligate intracellular spore-forming parasites that infect both animals and humans. To date, it was reported that there are more than 200 genera and nearly 1500 species [1] and 17 of these species have been reported to cause infection in humans [2]. One of these 17 species, *Enterocytozoon bieneusi* (*E. bieneusi*), is the most common species detected in humans which leads to opportunistic infections resulting with malabsorption and diarrhea [2]. Pulmonary and systemic disseminated microsporidiosis caused by *E. bieneusi* can result with death in immunocompromised patients [3, 4].

Various diagnostic methods have been used to identify *E. bieneusi*, including light microscopy, transmission electron microscopy, immunodetection using monoclonal antibodies, and PCR [1]. Nowadays, Real Time PCR with higher sensitivity and specificity is preferred for the diagnosis and epidemiological investigation of *E. bieneusi* [5]. Among these methods, only PCR, together with sequencing of ITS (ribosomal internal transcribed spacer) region of the ribosomal DNA, can give us information about the genotype profile of *E. bieneusi* [1]. Currently, phylogenetic analyses of ITS region identified 11 different groups (Groups 1–11). Group 1 containing 314 genotypes is divided into 9 subgroups (designated as 1a-1i). Within group 1, D, EbpC and type IV are the most frequently detected genotypes in humans and animals. Group 2 has 3 subgroups (2a to 2c). Groups between 3 and 11 contain fewer genotypes and are not divided into subgroups [6]. Approximately 90% of human pathogenic genotypes was reported to belong to Group 1 or Group 2 [7–9].

Although, D and Type IV, ERUSS1, and BEB6 as well as new genotypes were identified in different animal species in studies conducted in Türkiye [10–12], there are several studies that investigated the prevalence of *E. bieneusi* in humans living in Türkiye but these studies do not provide any information about the genotype profile of *E. bieneusi* [13–15]. Moreover, a study reported that prevalence of microsporidia (including *Encephalitozoon hellem/intestinalis*, *Encephalitozoon cuniculi*, *Encephalitozoon* sp., *E. bieneusi*) in colon cancer patients was 41,3% whereas the prevalence in healthy controls was %0 [16]. The finding indicates the importance of microsporidia infection including *E. bieneusi* in cancer patients. Therefore, in this study, we aimed to investigate the prevalence of *E. bieneusi* in three different groups. The first group consisted of patients with malignant solid tumors receiving chemotherapy and presenting with diarrhea. The second group consisted of patients with only diarrhea and the last group consisted of patients without diarrhea. The presence of *E. bieneusi* DNA was investigated by a Real Time PCR method with highly sensitivity and specificity which is developed by Verweij et al., (2007) and also used in our previous study [5, 17]. Later, *E. bieneusi*-positive samples were genotyped by sequencing ITS region amplified by nested PCR.

## Methods

### Patients

The stool samples belonging to 94 patients with malignant solid tumors receiving chemotherapy and presenting with diarrhea were collected and DNA extraction was performed in our previous study [18]. Among these patients, CD4^+^ cell count was between 200 and 400 cells/mm^3^, 400–800 cells/mm^3^ and 800–1500 cells/mm^3^. CD4^+^ cell counts were taken from oncology polyclinics that followed these patients [18]. All patients included in the study were outpatients. The stool samples belonging to control groups were collected in this study. The control groups contained patients with normal immune system presenting with only diarrhea symptom (*n* = 50) and patients with normal immune system without diarrhea (*n* = 50). After the DNA was isolated from control group stool samples using a commercial DNA isolation kit (Hipure stool DNA Kit) in accordance with the manufacturer’s protocol, these DNA samples were analyzed.

### Real time PCR

During Real Time PCR, a plasmid developed in our previous study and called pCR 2.1-ITS was used as positive control as well as to determine the analytical sensitivity of Real Time PCR and parasite load in each patient’s DNA analyzed [17]. The Real Time PCR standard curve was constructed using serially diluted positive controls (10^6^, 10^5^, 10^4^, 10^3^, 10^2^, 10^1^ and 10^0^/reaction) [17] and recalled in this study as the external standard curve using LightCycler software Version 4.0. The cycle threshold (Ct) values were between 18.32 (± 0.23) and 37.86 (± 1.18) for serially diluted positive controls. As the Ct values of serially diluted positive control plasmids and negative control were examined, the analytical sensitivity of Real Time PCR for the detection of *E. bieneusi* was determined as ≤ 1 copy plasmid/reaction and Ct values ≥ 36 were considered negative. Briefly, Real Time PCR method targeting ITS region of *E. bieneusi* was applied to DNA samples isolated from stool samples of patients as previously described [5]. The forward primer 5′-TGTGTAGGCGTGAGAGTGTATCTG-3′ and reverse primer 5′-CATCCAACCATCACGTACCAATC-3′ were used for amplifying ITS region. The hydrolysis probe used was FAM-5′-CACTGCACCCACATCCCTCACCCTT-3′-BHQ1. Each 20 µl reaction mix contained 4 µl 5x Taqman mix (Roche, Germany), 0.8 µl from each primer (2 µM), 1 µl probe (2 µM), 5 µl template DNA sample or control plasmid diluted, and 8,4 µl distilled water. PCR amplification reactions were performed using the following calculated control protocol: 15 min preincubation step at 95 °C, followed by 50 cycles of 15 s at 95 °C, 30 s at 60 °C and 30 s at 72 °C. As positive control, pCR 2.1-ITS plasmid (10^6^ to 10^0^/reaction) and one negative control which is prepared by distilled water were used. Quantification analysis for each sample was performed by 1.5 LightCycler Real Time instrument using LightCycler software, Version 4.0 (Roche, Germany).

### Genotyping

During genotyping, a nested PCR targeting the ITS region of *E. bieneusi* (~ 390 bp in length) was applied to Real Time PCR positive samples, as previously described [19]. Briefly, EBITS3 and EBITS4 primer pairs were used in the first round of PCR whereas in the second round of PCR, EBITS1 and EBITS2.4 primer pairs were used. For the first round preformed with 100 µl of PCR reaction, 2 µl template DNA, 10 µl Taq Buffer, 0,5 µl Taq DNA polymerase (GenTaq, 5 U/µl), 2 µl from each primer (10 µM), 16 µl 25 mM MgCl_2_, 2 µl 10 mM dNTP and 65,5 µl distilled water were used. Nested PCR was applied using the following calculated-control protocol: 5 min initial denaturation step at 94 °C, followed by 35 cycles of 30 s at 94 °C, 30 s at 57 °C, and 40 s at 72 °C, and a final extension of 10 min at 72 °C. In the second round, unlike the first round of PCR, 4 µl of amplified PCR product was used as template. Second round of nested PCR was performed using the following calculated-control protocol: 5 min initial denaturation step at 94 °C, followed by 30 cycles of 30 s at 94 °C, 30 s at 55 °C, and 40 s at 72 °C, and a final extension of 10 min at 72 °C.

To detect the genotype profiles of *E. bieneusi*, amplicons were run on 1% agarose gel, purified by the Qiaquick PCR purification kit (Qiagen, USA), and then sequenced. For sequencing, forward primer (EBITS1) which belongs to the second round of PCR was used. During the construction of phylogenetic tree, all sequence data obtained from this study were aligned with reference sequences with known genotype deposited in the NCBI (https://www.ncbi.nlm.nih.gov/) using the MEGA X [20], and a phylogenetic tree was created by MrBayes v.3.2.3 using Monte Carlo Markov Chain (MCMC) and Bayesian methods [21, 22]. Also, FigTree v.1.4.4 was used to visualize the phylogenetic tree [23].

### Statistical analysis

Molecular detection rates between the patient groups as well as its association with sex, age and residence were computed, and the statistical importance of the obtained values was detected by the independent samples t test using the GraphPad Prism. Statistically significant differences were determined at *P* < 0.05.

## Results

**Fig. 1:**
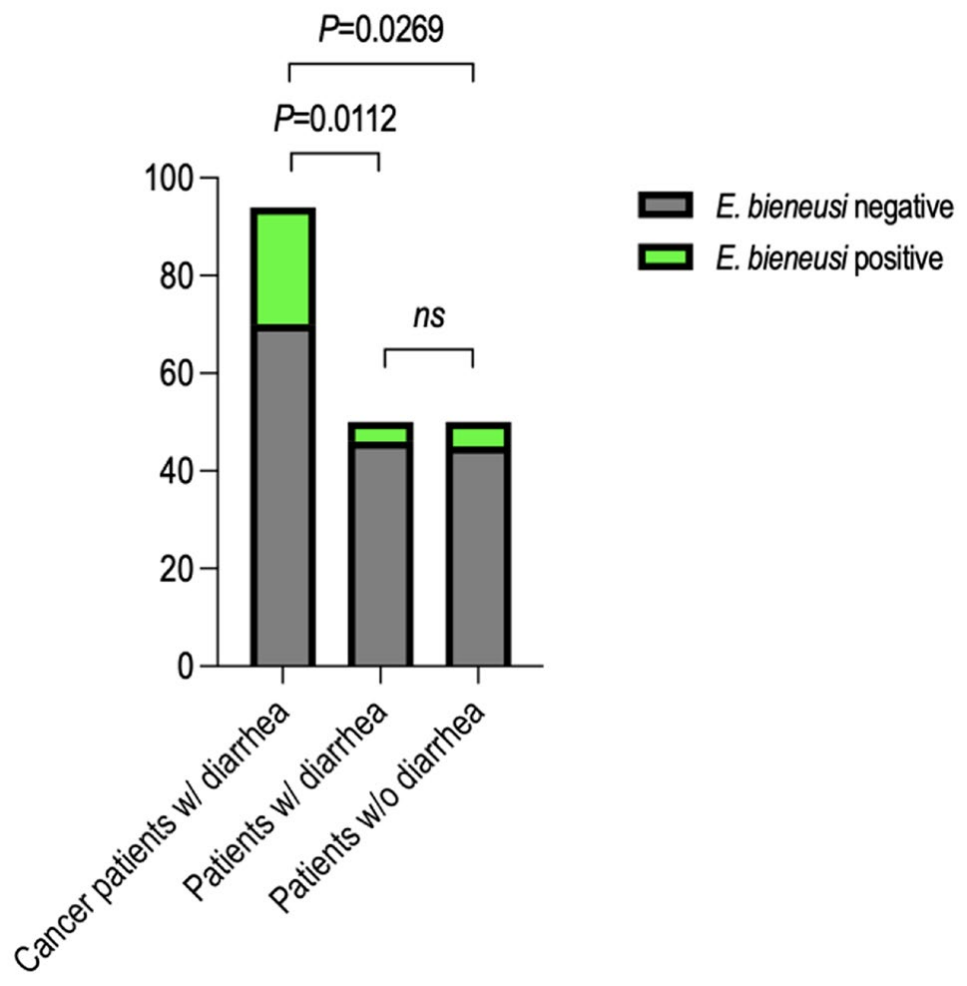
Comparison of the molecular prevalence values detected among different patient groups. Accordingly, molecular prevalence values of 25,5%, 8%, and 10% were detected among cancer patients with diarrhea, patients with diarrhea, and patients without diarrhea, respectively. The higher prevalence value detected in cancer patients with diarrhea was also statistically significant when compared to patients with diarrhea and patients without diarrhea

A total of 194 patients were screened by Real Time PCR for the presence of *E. bieneusi* and 33 of them were found to be *E. bieneusi*-positive. Accordingly, the molecular prevalence of *E. bieneusi* was 17% (33/194) among all patients analyzed. As the patients were categorized, the molecular prevalence of *E. bieneusi* increased to 25,5% (24/94) among patients with malignant solid tumors receiving chemotherapy and presenting diarrhea (Table 1). The molecular prevalence of *E. bieneusi* was found to be lower in patients with presenting only diarrhea (8%; 4/50) and patients without diarrhea (10%; 5/50). The Ct values among positive samples varied between 19,06 and 35,6 and the median Ct value was 34,46. The highest molecular prevalence value detected in patients with malignant solid tumors receiving chemotherapy and presenting diarrhea was also statistically significant compared to other control groups (P = 0,00112 and P = 0,0269) (Fig. 1). When all patients were classified as 200–400 cells/mm^3^, 400–800 cells/mm^3^ and 800–1500 cells/mm^3^ according to CD4^+^ cell count regardless of cancer type, the prevalence rates were 16,6% (1/6), 30,5% (22/72) and 6,25% (1/16), respectively. In addition, the relationship between sex, age, place of residence and *E. bieneusi* prevalence was given in Table 2.

**Fig. 2:**
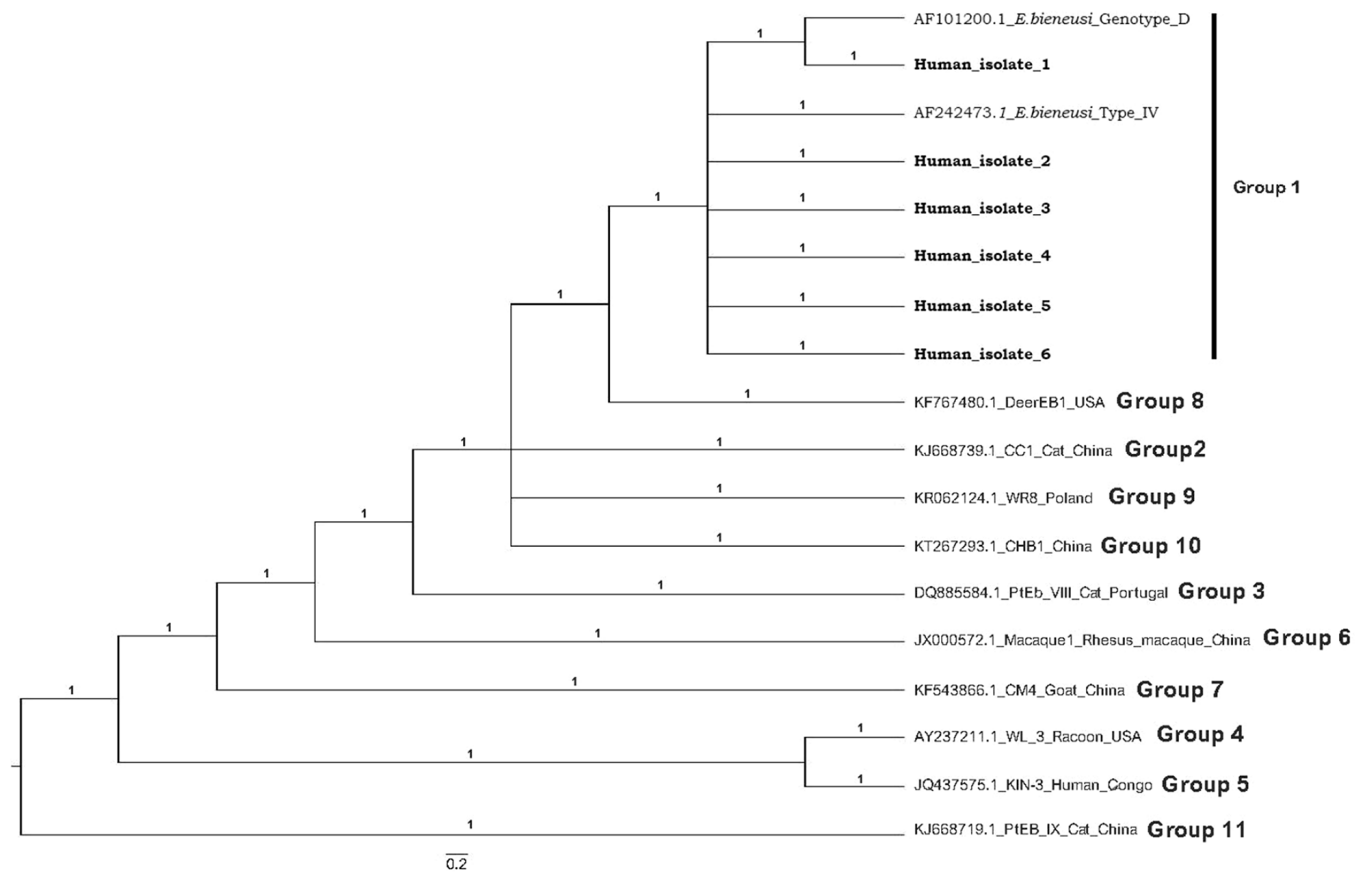
The phylogenetic analysis shows that *E. bieneusi* isolates detected in this study were grouped with refence sample (AF101200) classified as D and reference sample (AF242473) classified as Type IV. Among *E. bieneusi* isolates that were genotyped, Human_isolate_1_2 and 3 were detected in cancer patients with diarrhea whereas Human_isolate_4 was detected in patients with diarrhea and Human_isolate_5 and 6 were detected in patients without diarrhea

Among the 33 Real Time PCR positive samples, 10 of them were amplified by nested PCR and the remaining 23 were not amplified. Among these 10 amplified samples, 6 of them were successfully genotyped. Three genotypes were detected in cancer patients with diarrhea, one genotype was detected from patients with diarrhea and two genotypes were detected from patients without diarrhea. According to the results of phylogenetic tree, only one sample was grouped with a reference sample (AF101200) classified as D while the remaining five were grouped with a reference sample (AF242473) classified as Type IV (Fig. 2). Cancer type and genotype results of *E. bieneusi* positive patients were given in Table 1.

**Table 1:** Cancer type, Real Time PCR, and genotype results of *E. bieneusi* positive patients

Cancer type	Number of patients	Number of Real Time PCR positive patients	Genotype*
Colon cancer	27	5	D
Lymphoma	11	1	
Bladder cancer	5	1	
Breast cancer	5	-	
Pancreatic cancer	5	-	
Stomach cancer	5	-	
Lung cancer	4	3	Type IV
Rectal cancer	4	3	Type IV
Basal cell carcinoma	3	1	
Multiple myeloma	3	-	
Ovarian cancer	3	1	
Acute myeloblastic leukemia	2	1	
Laryngeal cancer	2	-	
Prostate cancer	2	2	
Renal cell carcinoma	2	1	
Uterine leiomyosarcoma	2	-	
Brain cancer	1	1	
Cervical cancer	1	-	
Endometrial cancer	1	-	
Esophageal cancer	1	1	
Hepatocellular carcinoma	1	-	
Malignant epithelial tumor	1	1	
Malignant mesenchymal tumor	1	-	
Plasmacytoma	1	1	
Skin cancer	1	1	

*Each genotype result belongs to only one patient in a group representing the type of cancer

**Table 2:** The relationship between sex, age, and place of residence and *E. bieneusi* prevalence

Parameters examined		Real Time PCR		Prevalence (%)	*P value*
		Positive	Negative		
Sex	Male (*n* = 107)	19	88	17,76	0,76
	Female (*n* = 87)	14	73	16,09	
Age	< 50 (*n* = 78)	8	70	10,26	0,08
	> 50 (*n* = 116)	25	91	21,55	
Residence	Urban (*n* = 127)	16	111	12,6	0,0243
	Rural (*n* = 67)	17	50	25,37	

## Discussion

Opportunistic parasitic infections are frequently asymptomatic or cause mild symptoms in immunocompetent individuals but can cause death in immunocompromised patients including cancer patients [24]. *E. bieneusi* is one of opportunistic parasites that can cause gastralgia, malabsorption, chronic diarrhea as well as pulmonary and systemic disseminated microsporidiosis in immunocompromised patients [2–4, 25]. In this study, because of its importance in immunocompromised patients, *E. bieneusi* was screened in patients with malignant solid tumors receiving chemotherapy and presenting diarrhea using Real Time PCR and a high molecular prevalence value of 25,5% was detected. To show the importance of *E. bieneusi* in cancer patients with diarrhea, patients with normal immune system presenting with diarrhea and without diarrhea also were screened for *E. bieneusi* and in these control groups, lower molecular prevalence values of 8% and 10% were detected, respectively. The higher molecular prevalence value detected in cancer patients with diarrhea was also statistically significant compared to other patient groups (Fig. 1). These results were remarkable in terms of showing the opportunistic nature of *E. bieneusi* in cancer patients. Moreover, in the previous studies, *Encephalitozoon* microsporidia was found to influence the immune response by controlling the apoptosis induction pathway and cell cycle, inhibiting the activation of the apoptotic protein Caspase-3 and the transcription of the universal protective tumor suppressor protein, p53. Also, *Encephalitozoon intestinalis* infection has been linked to increased host cellular mutation rates in mice that suggests a potential association between microsporidiosis and cancer induction [16, 26, 27].

The molecular prevalence value obtained from cancer patients with diarrhea was higher than previous study screening cancer patients under chemotherapy in Türkiye. The previous study reported that the prevalence of *E. bieneusi* was 9,7% in cancer patients under chemotherapy and 3,3% in control group using a commercial immunofluorescence antibody test [14]. It was thought that the prevalence difference may be due to the diagnostic approaches since Real Time PCR is more sensitive than fluorescence microscopy. A lower prevalence value of 4% for *E. bieneusi* in bone marrow transplant patients was also reported using a commercial immunofluorescence antibody test [13]. In another study conducted in Türkiye, the prevalence of *E. bieneusi* was reported as 9,1% in patients with diarrhea using multiplex nested PCR method [15]. Outside Türkiye, the prevalence of *E. bieneusi* was investigated in cancer patient living in China, Mexico, and Iran. Accordingly, in China, the prevalence of *E. bieneusi* was reported to be 4,3% in cancer patient with diarrhea while 0,8% in cancer patient without diarrhea using PCR [28]. In Mexico, the prevalence of *E. bieneusi* was reported to be 40% in pediatric patients with cancer using PCR [29]. In Iran, the prevalence of *E. bieneusi* was reported to be 1,7% in immunocompromised patients using PCR [30].

In this study, although not every *E. bieneusi* positive sample detected by Real Time PCR could be amplified by nested PCR, which has lower sensitivity than Real Time PCR, 10 of the Real Time PCR positive samples were amplified and 6 of them were successfully genotyped. According to the genotyping results, D and Type IV were identified in which D was identified in only cancer patients whereas Type IV was identified in three patient groups. The presence of D and Type IV in patients is not a surprising result as these are among the most frequently found genotypes in humans as well as animals [6]. For example, a study conducted in Iran also reported the presence of D as well as genotype E in cancer patients [30]. In another study conducted in China, the presence of D as well as a novel genotype named as HLJ-CP1 was reported in cancer patients [28]. Moreover, D and Type IV were also identified in stray cats living in İzmir in our previous study, indicating that these genotypes are circulating between humans and cats based on their zoonotic characteristics [12].

## Conclusion

In this study, detection of high molecular prevalence value of 25,5% by Real Time PCR indicates the importance of screening *E. bieneusi* in cancer patients with diarrhea. Also, *E. bieneusi*-positive samples isolated from patients analyzed were genotyped for the first time in Türkiye and D and Type IV were identified. As D and Type IV were also identified in stray cats in our previous study, the genotyping results obtained from humans in this study indicate that these genotypes are circulating between humans and cats.

## Data Availability

Accession numbers given by NCBI (https://www.ncbi.nlm.nih.gov/) for E. bieneusi isolates detected in this study are PP074316-PP074321.
